# Increased blood-based intratumor heterogeneity (bITH) is associated with unfavorable outcomes of immune checkpoint inhibitors plus chemotherapy in non-small cell lung cancer

**DOI:** 10.1186/s12916-022-02444-8

**Published:** 2022-07-29

**Authors:** Juan Zhou, Minwei Bao, Guanghui Gao, Yiran Cai, Lihong Wu, Lei Lei, Jing Zhao, Xianxiu Ji, Ying Huang, Chunxia Su

**Affiliations:** 1 Department of Oncology, Shanghai Pulmonary Hospital & Thoracic Cancer Institute, Tongji University School of Medicine, Shanghai, 200043 China; 2 Burning Rock Biotech, Guangzhou, China

**Keywords:** Non-small cell lung cancer (NSCLC), Immune checkpoint inhibitors (ICIs) plus chemotherapy, Blood-based intratumor heterogeneity (bITH), *LRP1B* mutation, Blood biopsy

## Abstract

**Background:**

The combination of immune checkpoint inhibitors (ICIs) and chemotherapy has been the standard first-line treatment for advanced non-small cell lung cancer (NSCLC) patients with driver-gene negative. However, efficacy biomarkers for ICIs-based combination therapy are lacking. We aimed to identify potential factors associated with outcomes of ICIs plus chemotherapy at baseline and dynamic changes in peripheral blood.

**Methods:**

We collected plasma samples of 51 advanced NSCLC patients without *EGFR/ALK/ROS1* alteration at baseline and/or after two treatment cycles of ICIs plus chemotherapy. A blood-based intratumor heterogeneity (bITH) score was calculated based on the allele frequencies of somatic mutations using a 520-gene panel. bITH-up was defined as a ≥ 10% increase in bITH score from baseline, with a second confirmatory measurement after treatment.

**Results:**

At baseline, the number of metastatic organs and lung immune prognostic index (LIPI) were significantly associated with shorter progression-free survival (PFS) of ICIs plus chemotherapy, while bITH and other common molecular biomarkers, including ctDNA level, blood-based tumor mutational burden (bTMB), and PD-L1 expression, had no effect on PFS. *LRP1B* mutation at baseline was significantly associated with favorable outcomes to ICIs plus chemotherapy. There were 37 patients who had paired samples at baseline and after two cycles of treatment, with the median interval of 53 days. Intriguingly, patients with bITH-up had significant shorter PFS (HR, 4.92; 95% CI, 1.72–14.07; *P* = 0.001) and a lower durable clinical benefit rate (0 vs 41.38%, *P* = 0.036) than those with bITH-stable or down. Case studies indicated that bITH was promising to predict disease progression.

**Conclusions:**

The present study is the first to report that increased bITH is associated with unfavorable outcomes of ICIs plus chemotherapy in advanced NSCLC patients.

**Supplementary Information:**

The online version contains supplementary material available at 10.1186/s12916-022-02444-8.

## Background

Immune checkpoint inhibitors (ICIs) targeting programmed cell death-1 (PD-1) and its ligand programmed cell death ligand-1 (PD-L1) have significantly improved long-term survival in PD-L1 selected patients with advanced non-small cell lung cancer (NSCLC) [1, 2]. However, PD-L1 ≥ 50% is observed below one third of patients and the efficacy of monotherapy for the whole population is far away from satisfactory [3]. Many studies have provided evidence that ICIs plus chemotherapy achieved higher efficacy and longer survival for unselected patients. According to a meta-analysis of KEYNOTE-024, KEYNOTE-042, KEYNOTE-189, and KEYNOTE-407 studies, even for newly diagnosed patients with PD-L1 ≥ 50%, pembrolizumab combined with chemotherapy further prolonged progression-free survival (PFS) than pembrolizumab monotherapy [4]. Nowadays, ICIs in combination with chemotherapy have been recommended as standard first-line therapy for advanced NSCLC patients without oncogenic driver alterations. Nevertheless, quite a large proportion of patients still suffer from treatment resistance, leading to an urgent need to search efficacy biomarkers for ICIs plus chemotherapy to avoid economic toxicity [5].

Multiple factors associated with clinical outcomes of immunotherapy are discovered such as PD-L1 expression, tumor mutation burden (TMB), gene expression profiling (GEP), and classification of tumor microenvironment [6]. However, majority of these current biomarkers are explored in monotherapy setting, and there is a lack of straightforward indicative biomarkers for maximizing efficacy of combination therapy. PD-L1 expression, which is the only comparable clinical predictor of a response to anti-PD-1/PD-L1 antibody monotherapy in patients with advanced NSCLC, remains to be related with clinical outcomes of ICIs-based combination therapy. The data from KEYNOTE-189 and KEYNOTE-407 studies showed that PD-L1 ≥ 50% was still associated with greater benefits with pembrolizumab-chemotherapy combination, but its predictive value was reduced as the survival benefits were significant across all PD-L1 tumor proportion score (TPS) groups, including patients with TPS < 1% [7].

Understanding biological processes of malignant tumor is critical for exploiting potential biomarkers to predict clinical benefits or resistance to therapy. Most tumors are complex ecosystems that would evolve continuously under selective pressure from their microenvironment exposed to physical, nutritional, and therapeutic perturbations, culminating with intratumor heterogeneity (ITH) [8]. ITH that embodied in both spatial and temporal dimensions can present as genomic [9], epigenomic [10], transcriptomic [11], proteomic [12], metabolomic [13], and tumor microenvironment heterogeneity [14], of which genetic ITH is more widely studied. Multi-region whole-exome sequencing (WES) on tumor tissues have revealed considerable spatial genome variation in the dimensions of intra-tumor, inter-metastases, and intra-metastases [15], which is increasingly recognized as a driver of tumor progression, drug resistance, and treatment failure in solid tumors. The TRACERx-Lung, currently the largest-scale study aimed to track genomic evolution of NSCLC, has systematically deciphered the genome heterogeneity of NSCLC [9], which has also brought unprecedent attention to ITH and propelled extensive researches. Recent studies have suggested that high level of ITH was associated with poor outcomes of anti-PD-(L)1 therapy in NSCLC [16]. However, ITH-based tissue is limited to be used in clinical practice due to the difficulty of multi-region sampling for advanced-stage patients and dynamic monitoring for treatment process. A previous study evaluated the potential application of circulating tumor DNA (ctDNA) in ITH analysis and suggested ctDNA profile maybe not an appropriate alternative because of the high missing rate of branch mutations [17]. However, theoretically, ctDNA provides a snapshot of overall genomic profiling for metastatic tumors, overcoming, to some extent, the bias from individual tumor biopsy. In addition, ctDNA enables real-time monitoring and tumor evolution trajectory capturing due to the convenience and non-invasive nature of blood sampling. Several biomarkers based on ctDNA detection have been established for cancer immunotherapy, such as maximum somatic allele frequency (MSAF) and blood-based tumor mutation burden (bTMB) [18, 19]. Therefore, developing better methods to evaluate ITH using ctDNA is of great value for understanding tumor evolution in clinical practice. Shannon Diversity Index (SDI), a formal diversity metric, is commonly used for tissue ITH evaluation and has been reported to be significantly associated with poor survival of immunotherapy in many cancer types [14, 16, 20]. Here, we utilized a weighted SDI suitable for blood to evaluate ITH and aimed to explore the association of blood-based ITH (bITH) and efficacy of ICIs plus chemotherapy in advanced NSCLC patients.

## Methods

### Patients’ enrollment and sample collection

We have collected whole blood samples from eligible patients at baseline and/or after two treatment cycles of ICIs plus chemotherapy. Patients who have advanced NSCLC with metastatic/recurrent or unresectable stages were enrolled into this study from Shanghai Pulmonary Hospital between September 2018 and January 2021. Eligible patients were as follows: (1) confirmed NSCLC by pathology, (2) staged IV or unresectable IIIB-IIIC according to the eighth edition of the TNM classification for lung cancer, (3) Eastern Cooperative Oncology Group (ECOG) performance status 0–2, (4) expected survival ≥ 3 months, (5) measurable lesions according to Response Evaluation Criteria in Solid Tumors version 1.1 (RECIST v1.1), and (6) received PD-1 inhibitor plus chemotherapy. Exclusion criteria included the following: (1) *EGFR/ALK/ROS1* alteration confirmed by amplification refractory mutation system-polymerase chain reaction (ARMS-PCR), (2) autoimmune diseases requiring systemic treatment within 2 years, (3) received other immunotherapy including but not limiting vaccines and adoptive cellular immunotherapy, (4) active multiple primary malignancies, and (5) receiving intensive immunosuppressive agents. Efficacy was evaluated according to RECIST v1.1. Objective response rate (ORR) was defined as the proportion of patients who had complete response and partial response (PR) to treatment. PFS was defined as the interval from the initiation of ICIs plus chemotherapy to confirmed disease progression or death of any cause. Similar to the concept of durable clinical benefits (DCB) promoted by previous studies [21], we defined DCB from ICIs-based combination therapy (DCBc) as PFS of at least 9 months and non-durable benefit from combination treatment (NDBc) as PFS < 9 months, with reference of median PFS in our cohort. The study was approved by ethics review board at Shanghai Pulmonary Hospital with reference number of L21-320 and was performed in accordance with the Declaration of Helsinki. At the time of sample collection, all participants provided written informed consents. The use of all samples in this study was also approved by International Cooperative Scientific Research on Human Genetic Resources (HGR) in China with reference number of 2021SQGH11583.

### DNA extraction and capture-based targeted DNA sequencing

DNA extraction and targeted sequencing were performed in Burning Rock Biotech, a commercial clinical laboratory which has demonstrated impressive performance in FDA-led Sequencing Quality Control Phase 2 (SEQC2) liquid biopsy program [22]. Briefly, 10 ml of venous blood was collected from patients, and peripheral white blood cells (WBCs) and plasma were separated by centrifugation at 1800×g for 10 min at 4 °C within 2 h after blood collection. Supernatant plasma was transferred to a new tube and centrifuged at 16,000×g for 10 min. WBCs were used for genomic DNA extraction as the germline controls. Circulating cell-free DNA (cfDNA) was extracted from plasma using QIAamp Circulating Nucleic Acid kit, according to the manufacturer’s standard protocol (Qiagen, Hilden, Germany). Fragments between 200 and 400 bp from the sheared genomic DNA and cfDNA were purified (Agencourt AMPure XP Kit, Beckman Coulter, CA, USA), hybridized with capture probes baits, selected with magnetic beads, and amplified. Target capture was performed using a commercial panel consisting of 520 genes (OncoScreen Plus®), spanning 1.86 megabases of the human genome. The quality and the size of the fragments were assessed by high sensitivity DNA kit using Bioanalyzer 2100 (Agilent Technologies, CA, USA). Indexed samples were sequenced on Nextseq 500 (Illumina, Inc., CA, USA) with paired-end reads and average sequencing depth of 1000× for WBCs and 10,000× for plasma samples.

### Sequence data analysis

Sequence data were mapped to the reference human genome (hg19) using Burrows-Wheeler Aligner version 0.7.10 [23]. Local alignment optimization, duplication marking, and variant calling were performed using Genome Analysis Tool Kit version 3.2 [24] and VarScan version 2.4.3 [25]. Plasma samples were compared against their own WBC control to identify somatic variants. Variants were filtered using the VarScan fpfilter pipeline; loci with depth less than 100 were filtered out. Base calling in plasma required at least 8 supporting reads for single nucleotide variations (SNVs) and 2 and 5 supporting reads for insertion-deletion variations (Indels), respectively. Variants with population frequency over 0.1% in the ExAC, 1000 Genomes, dbSNP, or ESP6500SI-V2 databases were grouped as single nucleotide polymorphisms (SNPs) and excluded from further analysis. Remaining variants were annotated with ANNOVAR (2016-02-01 release) [26] and SnpEff version 3.6 [27].

TMB was defined as the SNVs and small Indels (fusions and CNVs were excluded) locating at the coding region and its 20-bp upstream/downstream region. For accurate TMB calculation, MSAF should be ≥ 1% for plasma samples. The total size of the coding region for estimating TMB is 1.003 Mb for the 520-gene OncoScreen Plus panel. TMB was calculated according the following equation:

$$\mathrm{TMB}=\frac{\mathrm{mutation}\ \mathrm{count}\ \left(\mathrm{except}\ \mathrm{for}\ \mathrm{CNV},\mathrm{SV},\mathrm{SNPs},\mathrm{and}\ \mathrm{hot}\ \mathrm{mutations}\right)}{1.003\ \mathrm{Mb}}$$

### Calculation of common biomarkers used in this study

We used a cut-point of ≥ 16 as bTMB-high as described in a previous study [28]. ctDNA positive was defined as somatic mutation detectable at baseline, while ctDNA negative was defined as somatic mutation undetectable at baseline. The MSAF was defined as the maximum allele frequency of SNV/Indel mutation and was used to estimate the amount of tumor fraction of cfDNA in the sample. The median values of MSAF and mean AF in ctDNA-positive population were selected as the cut-points of MSAF and mean AF at baseline, respectively. ctDNA clearance was defined as mutation undetectable after treatment as previously reported [29]. MSAF drop was defined as a > 50% decrease in mutant allele fraction from baseline, with a second confirmatory measurement [18]. bTMB decrease was defined as the change of bTMB values < 0. Immunohistochemistry (IHC) staining of PD-L1 was performed using PD-L1 antibody (E1L3N or 22C3), and PD-L1 positive was defined as the TPS of PD-L1 ≥ 1%. The upper limit of normal (ULN) for lactate dehydrogenase (LDH) and C-reactive protein (CRP) are 246 IU/L and 10 mg/L, respectively. LDH-high and CRP-high were defined as greater than ULN of them. The cutoff for derived neutrophils/(leukocytes minus neutrophils) ratio (dNLR) was 3. The lung immune prognostic index (LIPI) was defined as combining dNLR greater than 3 and LDH greater than ULN and was classified into 3 groups (good, 0 factor; intermediate, 1 factor; poor, 2 factors) as described in a previous study [30].

### Calculation of bITH score using blood biopsy sequencing data

SDI was used for tissue ITH evaluation and was calculated as previously reported [20]. Given that the fraction of ctDNA in cfDNA can be characterized with MSAF, variant allele frequencies (VAFs) were firstly scaled with MSAF, and the resulting MSAF-corrected VAFs (MCVs) were then divided into ten equally sized bins. SDI was first introduced for biodiversity measurement, in which situation different regions of community were taken with equal importance. However, as tumor evolution theory suggested, the number of allelic copies of alterations was associated with its clonality [31]. Thus, the alterations with different relative allele frequencies should not be treated equally. Therefore, bITH score was subsequently calculated by introducing a weight function φi to the SDI formula to assign higher scores for samples with more diverse and dispersed clonality relationship per the following formula:

$$\mathbf{bITH}=-\sum \limits_{i=1}^n{\varphi}_i\ast {P}_i\mathit{\ln}\left({P}_i\right)$$

where *P*_*i*_ is the fraction of genomic alterations with MCVs in the *i*th bin relative to all alterations, and weight function φi is the median MCV of each bin.

Patients were divided into two groups based on the median value of baseline bITH scores in ctDNA positive population to examine the relationship of bITH score at baseline and PFS. Cumulative frequency plot of the change of bITH score was used to select the cutoff of bITH change.

### Statistical analysis

All statistical analyses and diagram drawing were performed using the R software (version 4.0.3). Wilcoxon rank sum test was conducted to examine the difference of bTMB distribution between two groups. Fisher’s exact test was used to compare the difference in population proportions of two groups. The Kaplan-Meier curve with log-rank test was used to examine the survival difference between two groups. Cox proportional hazards regression analysis was conducted to examine the relationships between PFS and multiple variables. Hierarchical clustering method was used to cluster somatic mutations detected at multiple time points. Survival (version 3.2-7) and survminer (version 0.4.8) packages in R software were used for survival analysis. *P* < 0.05 was considered statistically significant.

## Results

### Patient characteristics and ctDNA detection

**Fig. 1:**
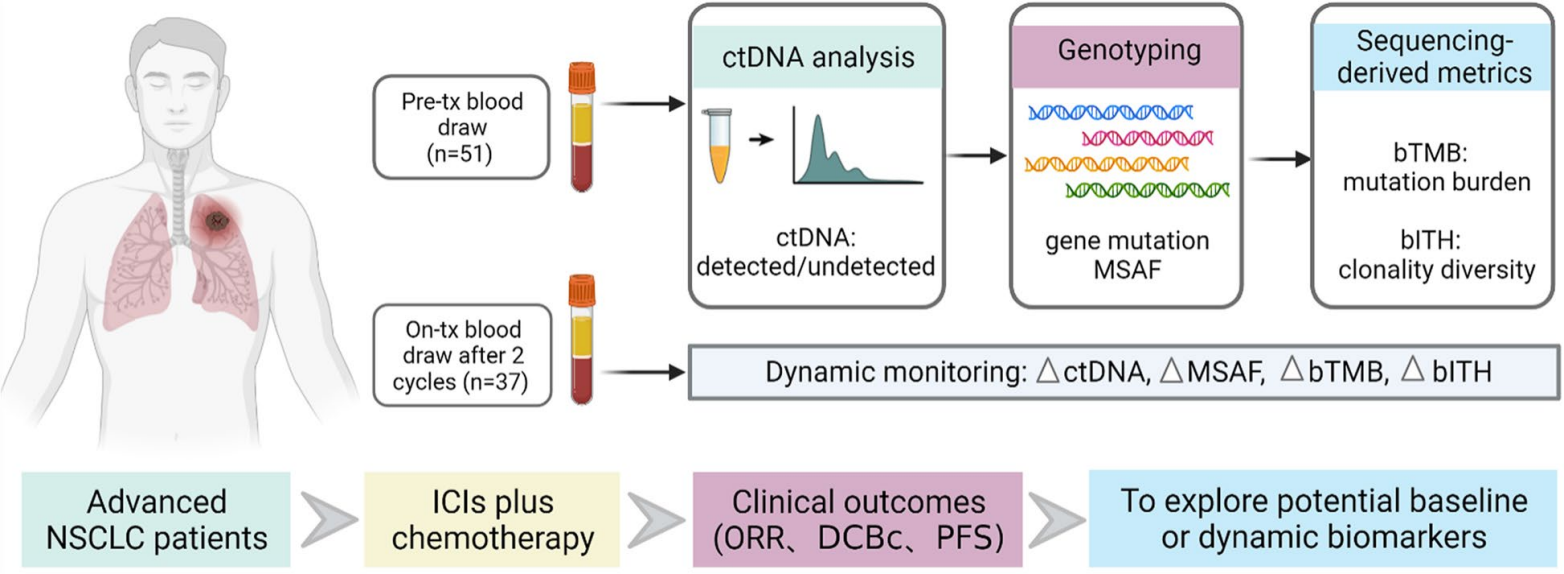
Flowchart of patient enrollment and study design

A total of 51 advanced NSCLC patients without sensitive *EGFR/ALK/ROS1* alterations were enrolled in the study (Fig. 1). The baseline characteristics of all participants are summarized in Table 1. Detailed information of clinical and molecular parameters is shown in Additional file 1: Table S1. Majority of patients were male (*n* = 40, [78.4%]), with a median age of 64 years. All patients except one were in good ECOG performance status (0–1). IHC staining of PD-L1 was tested in 30 patients, of whom 12 (40%) were PD-L1 positive. Most of patients received anti-PD-1 antibodies plus chemotherapy as the first-line treatment (*n* = 34, [66.7%]). In the whole cohort, the combination therapy achieved an ORR of 49.02% (25/51), a DCBc rate of 29.41% (15/51), and a median PFS of 9.37 months (Additional file 2: Fig. S1). We analyzed a total of 90 serial plasma samples that were obtained at baseline, and/or after two treatment cycles, and/or after disease progression. There were 37 patients who had paired samples at baseline and after two treatment cycles, with the median interval of 53 days. The baseline characteristics of these 37 patients were consistent with those of the whole study population (Table 1). Detailed dynamic data in these 37 patients are provided in Additional file 1: Table S2. The individual gene mutation list of all samples in this study are provided in Additional file 1: Table S3.

**Table 1:** Clinical characteristics of patients

Characteristics	With baseline biopsy (***n*** = 51)	With follow-up biopsy (***n*** = 37)
**Age, years**		
Median (range)	64 (46–76)	64 (46–72)
**Sex**		
Male	40 (78.4)	31 (83.8)
Female	11 (21.6)	6 (16.2)
**Smoking**		
Never	27 (52.9)	18 (48.6)
Ever/current	24 (47.1)	19 (51.4)
**Histology**		
NOS	7 (13.7)	6 (16.2)
Adenocarcinoma	27 (52.9)	17 (45.9)
Squamous	17 (33.3)	14 (37.8)
**Stage**		
IIIB-IIIC	13 (25.5)	10 (27.0)
IV	38 (74.5)	27 (73.0)
**Tumor size**		
≤ 5 cm	36 (70.6)	27 (73.0)
> 5 cm	15 (29.4)	10 (27.0)
**Number of metastatic organs**		
0–1	37 (72.5)	27 (73.0)
≥ 2	14 (27.5)	10 (27.0)
**Metastasis sites**		
Lung	26 (51.0)	18 (48.6)
Brain	4 (7.8)	2 (5.4)
Bone	8 (15.7)	6 (16.2)
Liver	2 (3.9)	2 (5.4)
Adrenal	2 (3.9)	2 (5.4)
Lymph gland	9 (17.6)	6 (16.2)
Others	0 (0)	5 (13.5)
**PD-L1**^**a**^		
Positive	12 (40.0)	9 (39.1)
Negative	18 (60.0)	14 (60.9)
Unknown	21	14
**ICIs Lines**		
1	34 (66.7)	26 (70.3)
≥ 2	17 (33.3)	11 (29.7)
**ICIs drug**		
Pembrolizumab	18 (35.3)	13 (35.1)
Camrelizumab	11 (21.6)	8 (21.6)
Sintinimab	16 (31.4)	11 (29.7)
Tislelizumab	4 (7.8)	4 (10.8)
Toripalimab	2 (3.9)	1 (2.7)
**Chemo-drug**^**b**^		
Pemetrexed	15 (29.4)	9 (24.3)
Gemcitabine	11 (20.6)	9 (24.3)
Paclitaxel	19 (37.2)	14 (37.8)
Docetaxel	6 (11.8)	5 (13.5)

*Abbreviation*: *NOS* not-otherwise-specified

^a^Antibodies for testing PD-L1 expression include E1L3N and 22C3

^b^All patients received platinum-based chemotherapy

### *LRP1B* mutation at baseline significantly associated with favorable outcomes of ICIs plus chemotherapy

In order to explore the potential efficacy-related biomarkers, we firstly dissected the relationship of clinical, biochemical and molecular characteristics at baseline with PFS using univariate COX regression analysis in all enrolled patients (Additional file 1: Table S4). We found that patients with no more than one organ involved distant metastasis had better PFS of ICIs plus chemotherapy than the others [hazard ratio (HR), 0.32; 95% CI, 0.15–0.67; *P* = 0.002], and there was no difference in PFS between patients stratified by other clinical characteristics, including age, sex, smoking status, histology, treatment line of ICIs plus chemotherapy and tumor size. For biochemical characteristics, the intermediate and poor LIPI was correlated with worse PFS of ICIs plus chemotherapy (HR, 2.73; 95% CI, 1.3–5.76; *P* = 0.006), while CRP level had no effect on PFS. For baseline molecular parameters, neither bITH nor other common biomarkers, such as PD-L1 expression, bTMB, MSAF, mean AF, and ctDNA detectable status, were associated with PFS of ICIs plus chemotherapy.

**Fig. 2:**
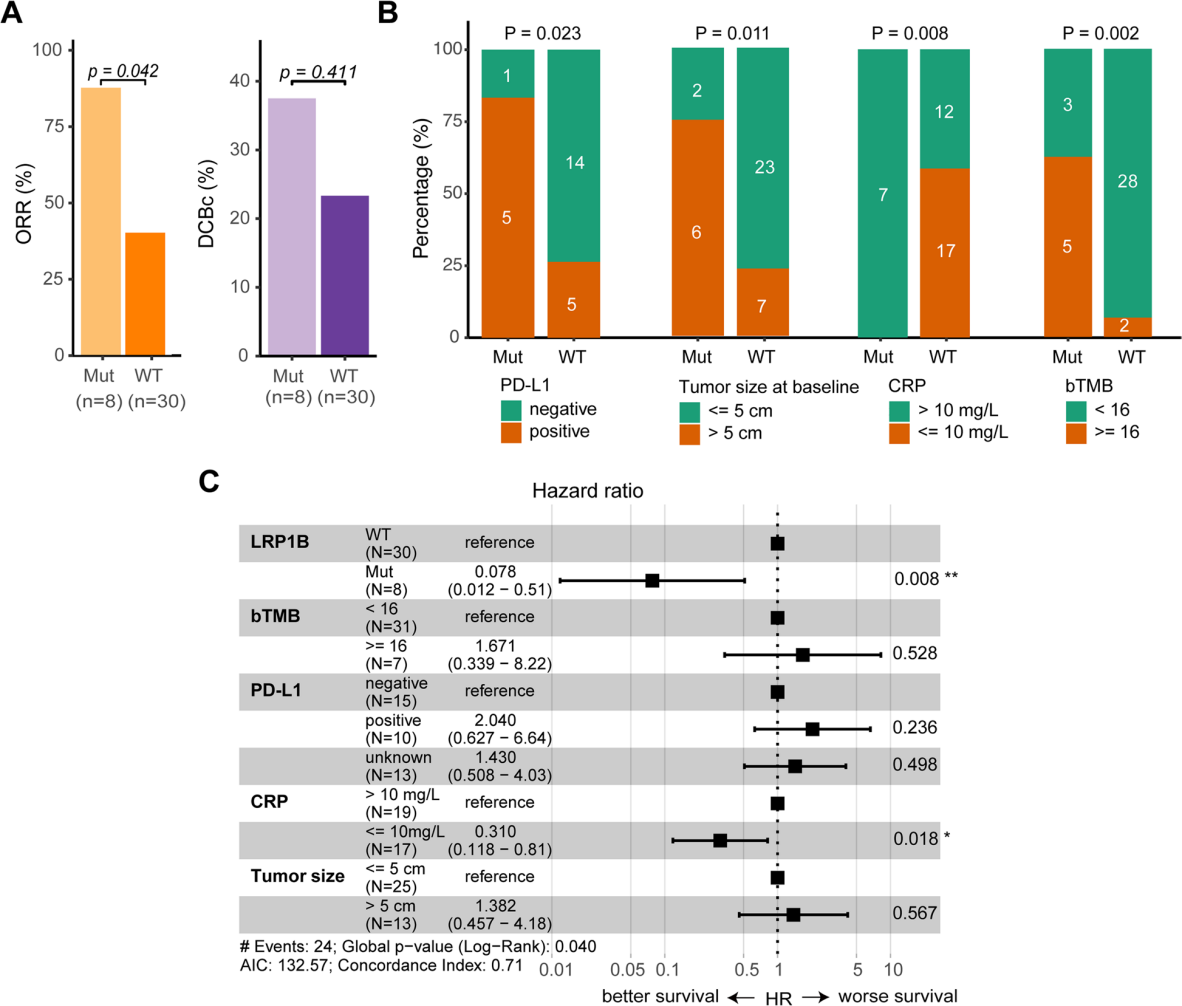
The association of *LRP1B* mutation at baseline with clinical outcomes of ICIs plus chemotherapy. **A** Objective response rate (ORR) (left) and durable clinical benefit (DCB) rate (right) between patients with *LRP1B* mutant and wild type. **B** The association of *LRP1B* mutation with PD-L1, tumor size at baseline, CRP level, and bTMB, respectively. Fisher’s exact test was used for statistical analysis. **C** Multivariate COX analysis of *LRP1B* mutation, bTMB, PD-L1, CRP, and tumor size at baseline. Mut, mutant; WT, wild-type; ORR, objective response rate; DCB, durable clinical benefit; CRP, C-reactive protein; bTMB, blood-based tumor mutational burden

We then investigated whether single gene mutation was correlated with clinical outcomes of ICIs plus chemotherapy in 38 patients who had detectable ctDNA mutations at baseline. First of all, given the clinical applicability of mutation frequency in populations and the statistical fluctuations of low-frequency mutations in small sample size cohort, only 19 genes with non-intron and nonsynonymous alteration frequency ≥ 4 (> 10% population mutation frequency in this cohort) were included in univariate analysis. As shown in Additional file 1: Table S5, 5 candidate genes with *P* value < 0.1 in univariate analysis were then included in a subsequent multivariate COX analysis to rule out the effects of co-mutations. *LRP1B* mutation and *KEAP1* mutation were significantly associated with PFS in multivariate analysis (*LRP1B* mutation: HR, 0.15; 95% CI, 0.03–0.72; *P* = 0.018; *KEAP1* mutation: HR, 4.26; 95% CI, 1.18–15.32; *P* = 0.027) (Additional file 1: Table S4). Patients with *LRP1B* mutation had higher ORR than those with *LRP1B* wild type, while there were no difference in ORR between *KEAP1* mutation and wild type (Fig. 2A and Additional file 2: Fig. S2A). In order to eliminate the influence of other clinical, biochemical and molecular characteristics, we also used Fisher’s exact test with *P* value < 0.1 to select covariates for multivariate analysis. We found that *LRP1B* mutation was associated with more PD-L1 positive, larger tumor size, higher CRP level, and bTMB value (Fig. 2B). Multivariate COX analysis showed that *LRP1B* mutation remained to be significantly associated with better PFS, which was independent of PD-L1, bTMB, tumor size, and CRP level (Fig. 2C). Similarly, *KEAP1* mutation was associated with higher bTMB, lower LIPI score and greater number of metastatic organs (Additional file 2: Fig. S2B). After multivariate correction for these three factors, *KEAP1* mutation was no longer an independent prognostic factor (Additional file 2: Fig. S2C). Overall, these data showed that *LRP1B* mutation at baseline was associated with favorable outcomes of ICIs plus chemotherapy.

### bITH-up significantly associated with unfavorable outcomes of ICIs plus chemotherapy

Next, we set out to determine the association of ctDNA-related dynamic changes after two cycles of treatment with clinical outcomes of ICIs plus chemotherapy in 37 patients who had paired samples. ctDNA clearance, MSAF drop, and bTMB decrease after treatment were reported to predict prolonged survival in patients treated with ICIs for NSCLC [18, 29, 32]. Unfortunately, in this study, no significant difference was found in PFS when stratified by these factors, regardless of whether the eight patients with ctDNA negative at baseline were excluded or not (Additional file 2: Fig. S3A-C).

**Fig. 3:**
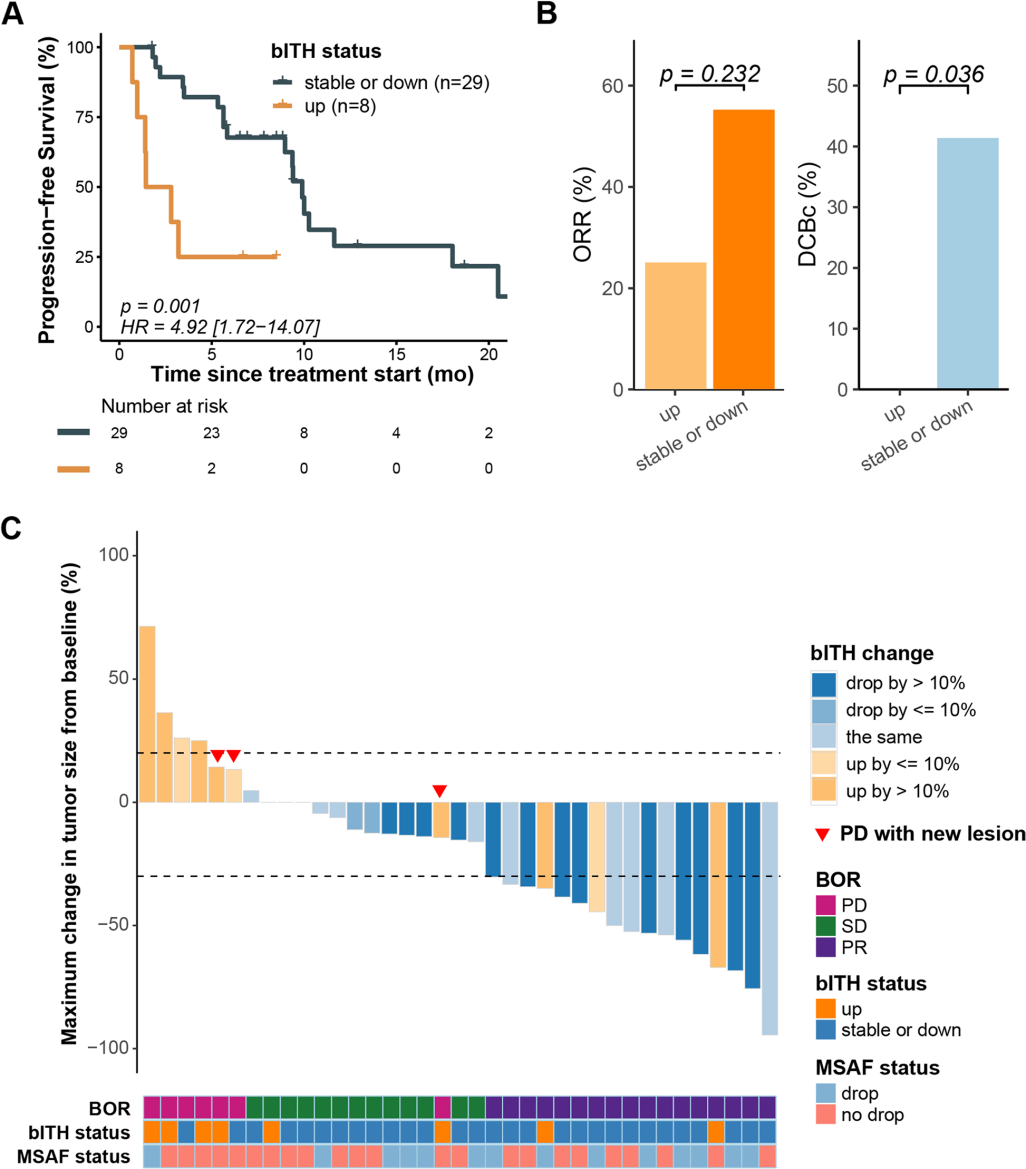
Association of the change of bITH scores after ICIs plus chemotherapy with clinical outcomes. **A** Kaplan-Meier curve for progression-free survival (PFS) according to bITH change status. bITH up was defined as a ≥ 10% increase in bITH score from baseline, with a second confirmatory measurement after treatment. Log-rank test was used for statistical analysis. **B** Objective response rate (ORR) (left) and durable clinical benefit (DCB) rate (right) among bITH up and bITH stable or down. Fisher’s exact test was used for statistical analysis. **C** Waterfall plot of bITH score change and the maximum change in tumor size from baseline. bITH, blood-based intratumor heterogeneity; HR, hazard ratio; ORR, objective response rate; DCB, durable clinical benefit; BOR, best overall response; PD, progressive disease; SD, stable disease; PR, partial response. MSAF, maximum somatic allele frequency

We first used the increase and decrease of bITH change to separate patients into two subgroups and found that patients with increased bITH score after two cycles of treatment had much worse PFS than those with decreased bITH score (HR, 3.46; 95% CI, 1.47–8.16; *P* = 0.003) (Additional file 2: Fig. S4A). To better determine an reasonable cut-off value for bITH change, we described the cumulative frequency plot of bITH change percentage from baseline to after two cycles treatment and found that 10% was the first elbow point and the percentage change increased slowly after 10% (Additional file 2: Fig. S4B). Considering that the increase of 0–10% may be attributed to slight disturbance from allele frequency variation of samples collected at two time points, we defined the increase of 0–10% as bITH-stable, the increase of ≥ 10% as bITH-up, and the others as bITH-down. Intriguingly, patients with bITH-up had significant shorter PFS (HR, 4.92; 95% CI, 1.72–14.07; *P* = 0.001) (Fig. 3A) than those with bITH-stable or down. Although patients with bITH-up had relatively lower proportion of ORR than those with bITH-stable or down without statistical significance, significant difference was observed in DCBc, as no patient with bITH-up achieved DCBc (0 vs 41.38%, *P* = 0.036) (Fig. 3B). To further exclude the influence of confounding factors, we compared the baseline characteristics between patients with bITH-stable or down and those with bITH-up and found all characteristics except treatment line of chemoimmunotherapy were balanced between the two groups (Additional file 1: Table S6 and Additional file 2: Fig. S5A). After being adjusted by the treatment line, multivariate analysis suggested that bITH-up remained still significantly associated with shorter PFS (Additional file 2: Fig. S5B).

Taken together, our data suggested that bITH-up after treatment was significantly associated with unfavorable outcomes of ICIs plus chemotherapy. Moreover, we found that all seven patients with progressive disease (PD) after two cycles treatment have increased bITH score, and 63.6% (7/11) of patients with increased bITH were confirmed PD (Fig. 3C), which suggested that the high potential value of bITH as a biomarker for forecasting the occurrence of PD.

### bITH outperforms MSAF in forecasting disease progression

**Fig. 4:**
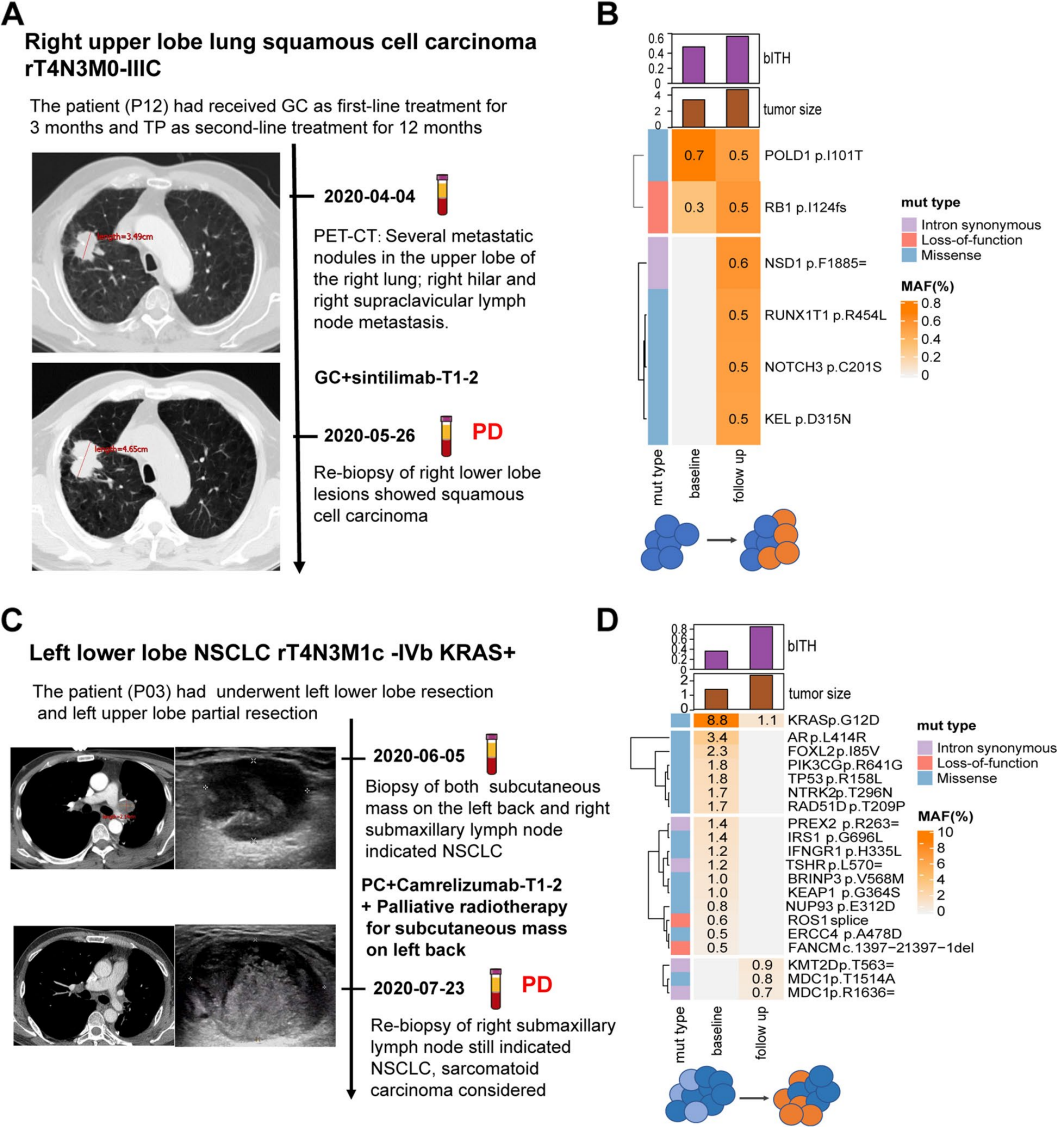
Case analysis of two patients with MSAF decreased but bITH up after treatment experienced disease progression as the best response to treatment. **A**, **C** The timeline, treatment history, and radiographic response to treatment of the patient P12 (**A**) and P03 (**C**). **B**, **D** The changes of bITH score, tumor size, and detected somatic mutations at baseline and after two cycles of therapy in patient P12 (**B**) and P03 (**D**). Hierarchical clustering method was used to cluster somatic mutations detected at two time points. bITH, blood-based intratumor heterogeneity; PD, progressive disease; MSAF, maximum somatic allele frequency; MAF, mutant allele frequency

The role of bITH change in forecasting the occurrence of PD was more obvious in typical cases. Patient P12, a 68-year-old male, was diagnosed with squamous NSCLC recurrence staged at rT4N3M0-IIIC after right upper lobe resection. He received gemcitabine plus sintilimab as the third-line therapy. However, his disease rapidly progressed after 1.4 months of treatment (Fig. 4A). Next-generation sequencing (NGS) detection showed that the bITH score increased by 28.7%, while MSAF decreased slightly after progression (Fig. 4B). Notably, the allelic frequency of the new mutations identified only in plasma after progression was equivalent to that of the pre-existing mutations at baseline, which meant that tumor might evolve a new subclonal, and there were at least two comparable populations of tumor cells after treatment.

Patient P03 was diagnosed with NSCLC recurrence staged at rT4N3M1c-IVb with *KRAS* mutation after left lower lobe resection and left upper lobe partial resection. He was confirmed to have a recurrence at right submaxillary lymph node, mediastinal lymph node, and subcutaneous mass on the left back 2 months after surgery. Then, the patient received cisplatin, paclitaxel plus camrelizumab regimen, and palliative radiotherapy for subcutaneous mass on the left back. However, the right submaxillary lymph node enlarged after 21 days of treatment, while subcutaneous mass and mediastinal lymph node shrunk (Fig. 4C). ctDNA data showed that MSAF and mutation counts dropped significantly after progression, but the bITH score increased by 133.88% (Fig. 4D). Although lots of mutations disappeared after treatment, new mutations occurred with the frequency comparable to that of *KRAS* missense mutation, which attributed to the increase of bITH score.

In these two cases, although the MSAF decreased, the bITH score increased, which meant the clone numbers and/or clone diversity increased, and the disease was confirmed to be progressed. Therefore, the increase of bITH score might be a better signal for disease progression than MSAF.

### bITH has the potential to predict disease progression prior to radiographic assessment

**Fig. 5:**
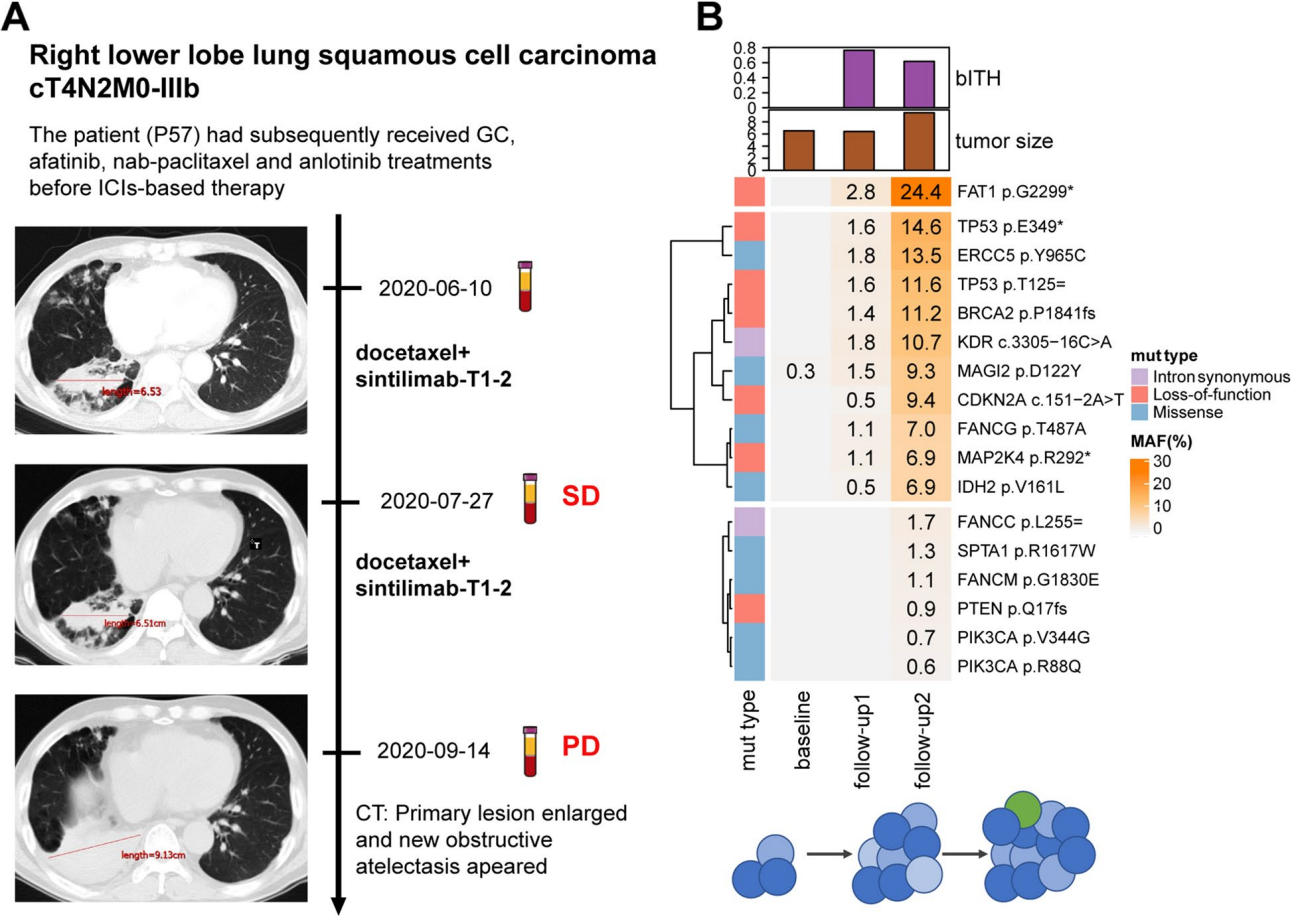
Case analysis of patient with dynamic ctDNA detection at baseline, after two cycles of treatment, and after disease progression. **A** The timeline, treatment history, and radiographic response to treatment of the patient P57. **B** The changes of bITH score, tumor size, and detected somatic mutations at baseline and after two cycles of therapy in patient P57. Hierarchical clustering method was used to cluster somatic mutations detected at multiple time points. bITH, blood-based intratumor heterogeneity; SD, stable disease; PD, progressive disease; MSAF, maximum somatic allele frequency; MAF, mutant allele frequency

Of note, there were 62.5% (5/8) of patients in bITH-up group who had radiological PD before the second blood collection, which reduced the predictive value of bITH. However, longitudinal monitoring at more time points showed the underlying role of bITH for predicting PD prior to radiographic assessment. Patient P57, whose disease was diagnosed as right lower lobe squamous NSCLC staged at cT4N2M0-IIIb, received docetaxel plus sintilimab regimen as the fifth-line therapy (Fig. 5A). He achieved stable disease response after two cycles of treatment, but the disease progressed after 3 months. ctDNA sequencing was performed on samples at baseline, after two cycles of treatment when he achieved SD (the first follow-up), and after disease progression (the second follow-up). Although the target lesion remained stable at the first follow-up, bITH score increased sharply when loss-of-function mutations of many tumor suppressor genes occurred, resulting a 49-day lead-time on detection of progression compared with the radiological assessment (Fig. 5B). These mutations still existed at the second follow-up with higher mutation allelic frequencies. Moreover, new mutations, such as two *PIK3CA* gain-of-function mutations and one *PTEN* loss-of-function mutation, appeared at the second follow-up. These new mutations, which were associated with the activation of PI3K-Akt signaling pathway, may contribute to drug resistance and tumor progression.

## Discussion

In this study, we investigated potential peripheral blood biomarkers associated with therapeutic efficacy of ICIs plus chemotherapy in advanced NSCLC patients without sensitive *EGFR/ALK/ROS1* alterations. Our data showed that (i) at baseline, the number of metastatic organs and LIPI index were significantly associated with poor PFS of ICIs plus chemotherapy, while bITH and other common molecular biomarkers, including ctDNA level, bTMB and PD-L1 expression had no effect on PFS; (ii) *LRP1B* mutation at baseline was significantly associated with favorable outcomes of ICIs plus chemotherapy; (iii) bITH-up after treatment was significantly associated with poor outcomes of ICIs plus chemotherapy; and (iv) case studies revealed that bITH outperformed MSAF in forecasting PD and had the potential for predicting PD prior to radiographic assessment.

Searching efficacy biomarkers has been the hotspot of immunotherapy research, and new challenges have been encountered in ICIs-based combination treatment setting. PD-L1 and TMB have been widely adopted in clinical practice to identify potential candidates for ICIs monotherapy, with the higher values predicting the better therapeutic efficacy. However, neither PD-L1 nor TMB could select patients who would benefit more from ICIs plus chemotherapy over chemotherapy alone, and the hazard ratios were even lower in patients with PD-L1 ≥ 50% or patients with TMB ≥ 10 mut/Mb but PD-L1 < 1% [7, 32, 33]. Our results reveal that these two baseline biomarkers are not associated with the efficacy of ICIs plus chemotherapy, which are consistent with previous reports [32]. In addition, many previous studies suggested that some very easy captured baseline biochemical indicators, including CRP, LDH, dNLR, and the combination model-LIPI, could also serve as biomarkers to predict survival benefits of ICIs monotherapy [30, 34], but their roles in the treatment setting of ICIs plus chemotherapy remained unclear. Our data suggested that LIPI could be used to distinguish potential beneficiaries, while CRP could not. The poor performance of these biomarkers in ICIs plus chemotherapy setting may be attributed to synergistic antitumor effects of the two treatments, as previous studies suggested that many chemo-drugs have immunomodulatory effects, such as release of antigens, maturation of dendritic cells, enhancement of antigen presentation, depletion of immune suppressive cells, and direct stimulation of T cells [35].

Although molecular biomarkers such as ctDNA status, MSAF, bTMB, and bITH at baseline failed to be associated with the efficacy of ICIs plus chemotherapy, we observed *LRP1B* mutation at baseline was significantly associated with favorable outcomes to ICIs plus chemotherapy. Several studies have suggested that *LRP1B* mutation was correlated with high TMB and improved outcomes with ICIs monotherapy in melanoma, NSCLC, and other solid tumors, which are consistent with our results in ICIs plus chemotherapy [36–40]. In our study, although patients with *LRP1B* mutation had higher bTMB, CRP, PD-L1 positive rate, and larger tumor size, the association of *LRP1B* mutation with improved outcomes to ICI plus chemotherapy remained consistent after controlling for these factors. Chen et al. investigated the potential mechanism behind *LRP1B* mutation and immune response in melanoma and NSCLC and found that cell cycle and antigen processing pathways were significantly altered in samples with *LRP1B* mutation, and patients with *LRP1B* mutation had higher T cell inflamed gene expression profiling scores [37]. Further mechanism studies in immunocompetent preclinical models of NSCLC with ICIs plus chemotherapy are needed to clarify the function of *LRP1B* mutation in immune response.

ITH based on tumor tissues is widely studied and has been associated with increased resistance to immunotherapy [41, 42]. Wolf et al. designed a novel melanoma mouse model to uncouple tumor mutational load and tumor heterogeneity and discovered that increased heterogeneity led to strong immunosuppressive tumor microenvironment to promote tumor growth. They also used SDI to quantify the genetic ITH and found that high ITH was significantly associated with poor survival in melanoma patients treated with ICIs [20]. Recently, in NSCLC, Fang et al. developed and validated an ITH index in tumor samples and found that ITH index was associated with the efficacy of immunotherapy. When applied to ctDNA data, however, ITH index did not perform well in differentiating immunotherapy efficacy [16]. Due to the limitation of the algorithm for calculating heterogeneity in blood test, no dynamic analysis has been reported on the correlation between heterogeneity change and immunotherapy, especially ICIs plus chemotherapy. The novel algorithm we developed to evaluate bITH was based on a weighted SDI, which considered both clone numbers and their genetic diversity. Although bITH score at baseline could not predict efficacy from ICIs plus chemotherapy, bITH-up (defined as ≥10% increase) after treatment was found to be significantly associated with poor outcomes of ICIs plus chemotherapy. Our work fills in the gaps of dynamic analysis of heterogeneity studies in blood test.

Two cases (P12 and P03) with MSAF decrease but bITH increase experienced disease progression soon after treatment, indicating that the information of tumor development reflected by bITH dynamics could not be reflected in the change of ctDNA levels. Moreover, all seven patients with PD after two cycles of treatment had increased bITH score, while only four patients had increased ctDNA level. Therefore, we believe that bITH score is superior to identify PD than ctDNA level. The increase of ITH based on multi-regions sampling in the same lesion is believed to reflect the subclonal mutation evolution inside the corresponding tumor [16, 17]. Our bITH score mirrored the diversity changes of tumor cell populations resulted from evolution of all tumors including primary and metastasis lesions, though it is hard to distinguish clonal and subclonal mutations. For example, in patient P03, although MSAF and mutation counts dropped significantly after progression, bITH score increased, which indicates the emergence of a population of resistant tumor cells even if the other populations do respond to treatment. Together, bITH should be considered as a great supplement to tissue to evaluate ITH and a promising biomarker to predict treatment resistance.

There are several limitations in our study. Firstly, it is a retrospective study. Due to the limitation of blood collection time, only patients having blood samples collected can be enrolled into this cohort. Besides, our cohort included different therapeutic regimens of ICIs plus chemotherapy, broughting biases and confusions that may have influenced our results. However, all ICIs used in our cohort are approved by national medical products administration (NMPA) of China and these treatment regimens reflected the real-world clinical practice in China. Secondly, the cohort includes limited samples. However, data maturity is relatively high in our cohort as thirty-one patients (60% of the cohort) were followed to the occurrence of the progression event. Thirdly, mutations with low variant allelic frequency in plasma are difficult to be detected due to the limitation of blood testing techniques. However, blood testing is non-invasive and can be used for dynamic monitoring. Fourthly, although mutations in the blood could not distinguish between clonal and subclonal mutations, the bITH algorithm we developed can still capture the overall tumor heterogeneity well, which overcomes bias from sampling on one tumor lesion for patients with many metastases to some extent. Finally, we did not validate our results in another cohort receiving ICIs plus chemotherapy, and more validations are needed in future.

## Conclusions

We used the modified SDI method to evaluate the feasibility of bITH in blood. Our study is the first to report that increased bITH is associated with poor outcomes to ICIs plus chemotherapy in advanced NSCLC patients.

## Supplementary Information

**Additional file 1: Table S1**. Clinical characteristics and molecular biomarkers at baseline in all enrolled patients. **Table S2**. Clinical characteristics and molecular biomarkers in patients with paired samples at baseline and after two treatment cycles. **Table S3**. The individual gene mutation list of all samples in this study. **Table S4**. Univariate COX analysis of biomarkers at baseline. **Table S5**. Gene mutations at baseline correlated with clinical outcomes of ICIs plus chemotherapy. **Table S6**. Comparison of clinical characteristics between patients with bITH-up and those with bITH-stable or down.

**Additional file 2: Fig. S1**. Clinical outcomes of ICIs plus chemotherapy in the enrolled cohort. **Fig. S2**. The association of KEAP1 mutation at baseline with clinical outcomes of ICIs plus chemotherapy. **Fig. S3**. Kaplan-Meier curve for progression-free survival (PFS) according to ctDNA clearance status, MSAF change status, and bTMB change status. **Fig. S4**. The cutoff selection of bITH change. **Fig. S5**. The association of bITH change with progression-free survival of ICIs plus chemotherapy after adjusting to the treatment line of chemoimmunotherapy.

## Data Availability

The datasets analyzed during the current study are available in Supplementary materials Additional file 1: Table S1-S3.

## Abbreviations

bITH: Blood-based intratumor heterogeneity
bTMB: Blood-based tumor mutational burden
cfDNA: Circulating cell-free DNA
CRP: C-reactive protein (CRP)
ctDNA: Circulating tumor DNA
DCB: Durable clinical benefits
DCBc: Durable clinical benefits from ICIs-based combination therapy
dNLR: Derived neutrophils/(leukocytes minus neutrophils) ratio
ECOG: Eastern Cooperative Oncology Group
GEP: Gene expression profiling
HGR: Human genetic resources
HR: Hazard ratio
ICIs: Immune checkpoint inhibitors
ITH: Intratumor heterogeneity
LDH: Lactate dehydrogenase
LIPI: Lung immune prognostic index
MCVs: MSAF-corrected VAFs
MSAF: Maximum somatic allele frequency
NDB: Non-durable benefit
NDBc: Non-durable benefit from combination treatment
NGS: Next-generation sequencing
NMPA: National medical products administration
NSCLC: Non-small cell lung cancer
ORR: Objective response rate
PD: Progressive disease
PD-1: Programmed cell death-1
PD-L1: Programmed cell death ligand 1
PFS: Progression-free survival
PR: Partial response
RECIST v1.1: Response Evaluation Criteria in Solid Tumors version 1.1
SD: Stable disease
SDI: Shannon diversity index
TMB: Tumor mutational burden
TPS: Tumor proportion score
ULN: The upper limit of normal
VAF: Variant allele frequency
WES: Whole-exome sequencing
