# Supplementary material for: Increased blood-based intratumor heterogeneity (bITH) is associated with unfavorable outcomes of immune checkpoint inhibitors plus chemotherapy in non-small cell lung cancer
Source: BMC Med. 2022 Jul 29;20:256. doi: 10.1186/s12916-022-02444-8 (PMC9335993; doi:10.1186/s12916-022-02444-8)

Additional file 2: Figures S1-S5

Fig. S1. Clinical outcomes of ICIs plus chemotherapy in the enrolled cohort.

(A-B) The percentage of patients with each best objective response (BOR) (A), and with durable clinical benefit (DCB) and non-durable benefit (NDB) (B) in the cohort.

(C) Progression-free survival (PFS) of this cohort.

BOR, best overall response; PD, progressive disease; SD, stable disease; PR, partial response; DCB, durable clinical benefit; NDB, non-durable benefit; PFS, progression-free survival.

Fig. S2. The association of *KEAP1* mutation at baseline with clinical outcomes of ICIs plus chemotherapy.

(A) Objective response rate (ORR) (left) and durable clinical benefit (DCB) rate (right) between patients with *KEAP1* mutant and wild type. (B) The association of *KEAP1* mutation with bTMB, LIPI and the number of metastatic organs, respectively. Fisher’s exact test was used for statistical analysis. (C) Multivariate COX analysis of *KEAP1* mutation, bTMB, LIPI and the number of metastatic organs.

Mut, mutant; WT, wildtype; ORR, objective response rate; DCB, durable clinical benefit; bTMB, blood-based tumor mutational burden; LIPI, lung immune prognostic index.

Fig. S3. Kaplan-Meier curve for progression-free survival (PFS) according to ctDNA clearance status in patients excluded ctDNA negative at baseline (A), in all patients (B), according to MSAF change status in all patients (C), according to bTMB change status in all patients (D). MSAF drop was defined as a >50% decrease in mutant allele fraction from baseline, with a second confirmatory measurement. bTMB change < 0 was defined as bTMB decrease, while the others were defined as bTMB increase. Log-rank test was used for statistical analysis.

HR, hazard ratio; MSAF, maximum somatic allele frequency; bTMB, blood-based tumor mutational burden.

Fig. S4. The cutoff selection of bITH change.

(A) Kaplan-Meier curve for progression-free survival (PFS) between increased bITH and decreased bITH subgroups. bITH change > 0 was defined as bITH increase, while the others were defined as bITH decrease. Log-rank test was used for statistical analysis. (B) Cumulative frequency plot of bITH change for the cutoff point selection. The dashed line corresponds to a bITH change percentage of 10%.

bITH, blood-based intratumor heterogeneity; HR, hazard ratio.

Fig. S5. The association of bITH change with progression-free survival of ICIs plus chemotherapy after adjusting to the treatment line of chemoimmunotherapy.

(A) The association of bITH change with treatment line. Fisher’s exact test was used for statistical analysis. (B) Multivariate COX analysis of bITH change and treatment line.

bITH, blood-based intratumor heterogeneity; HR, hazard ratio.

Fig. S1


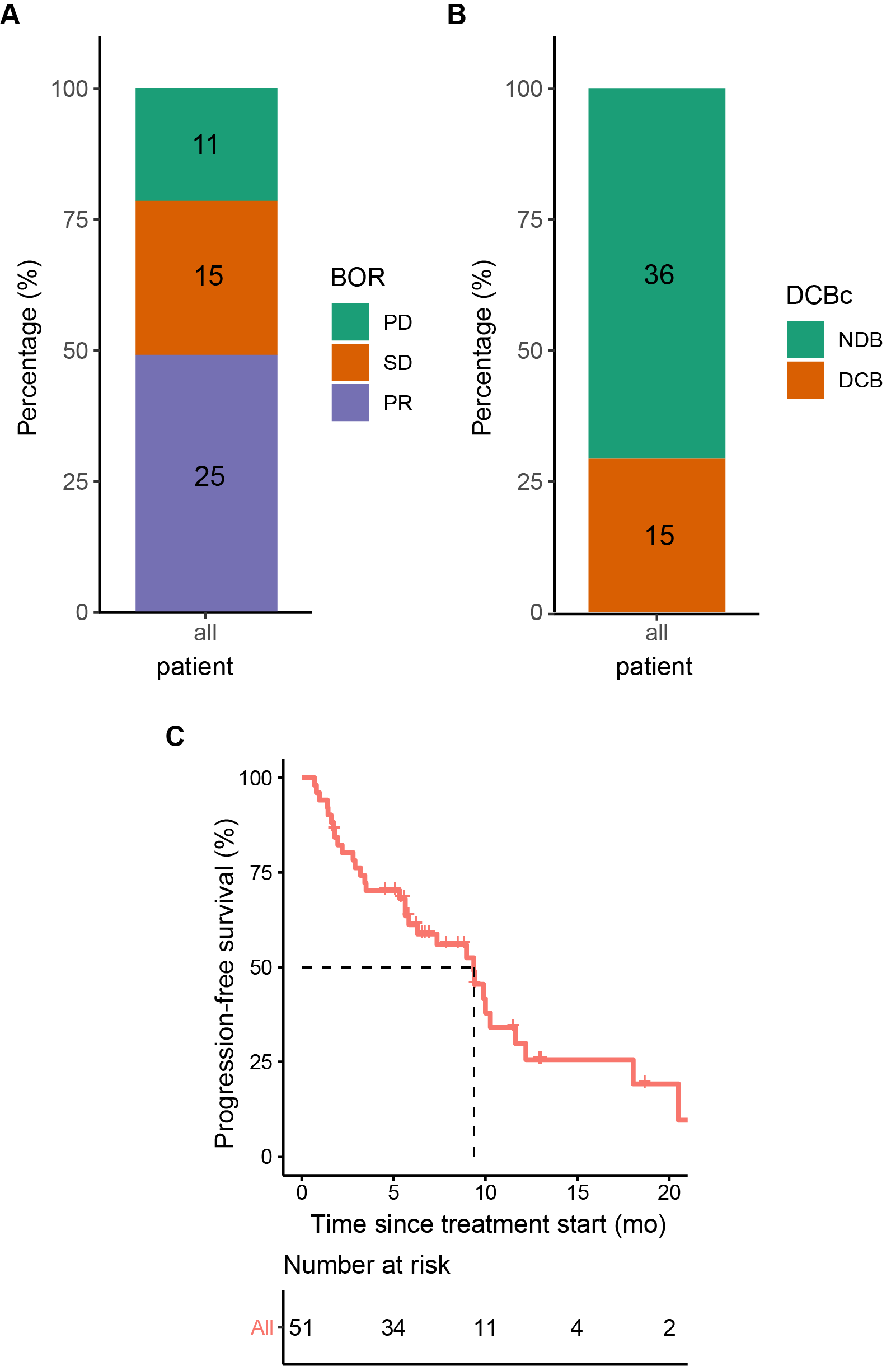


Fig. S2


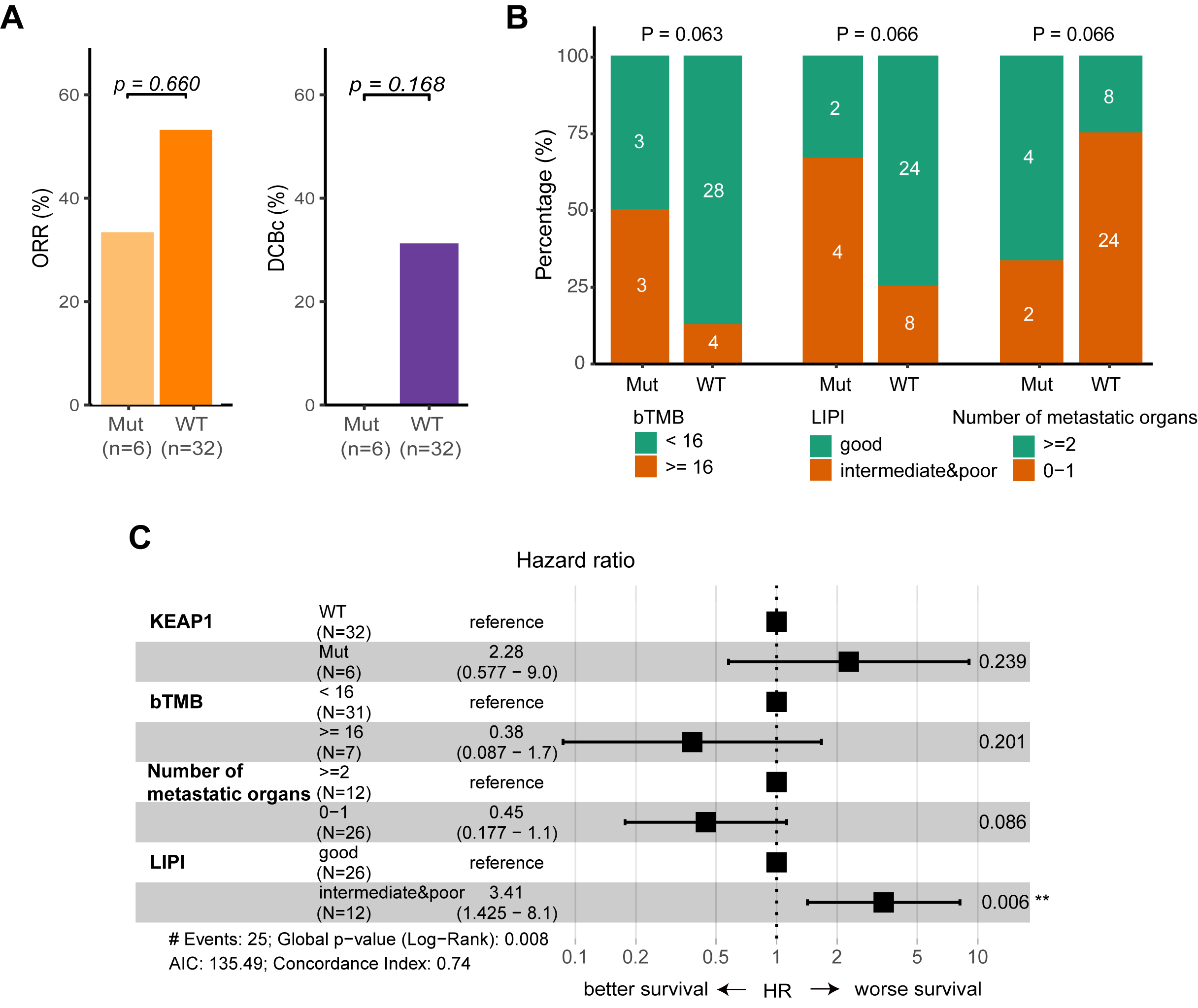


Fig. S3


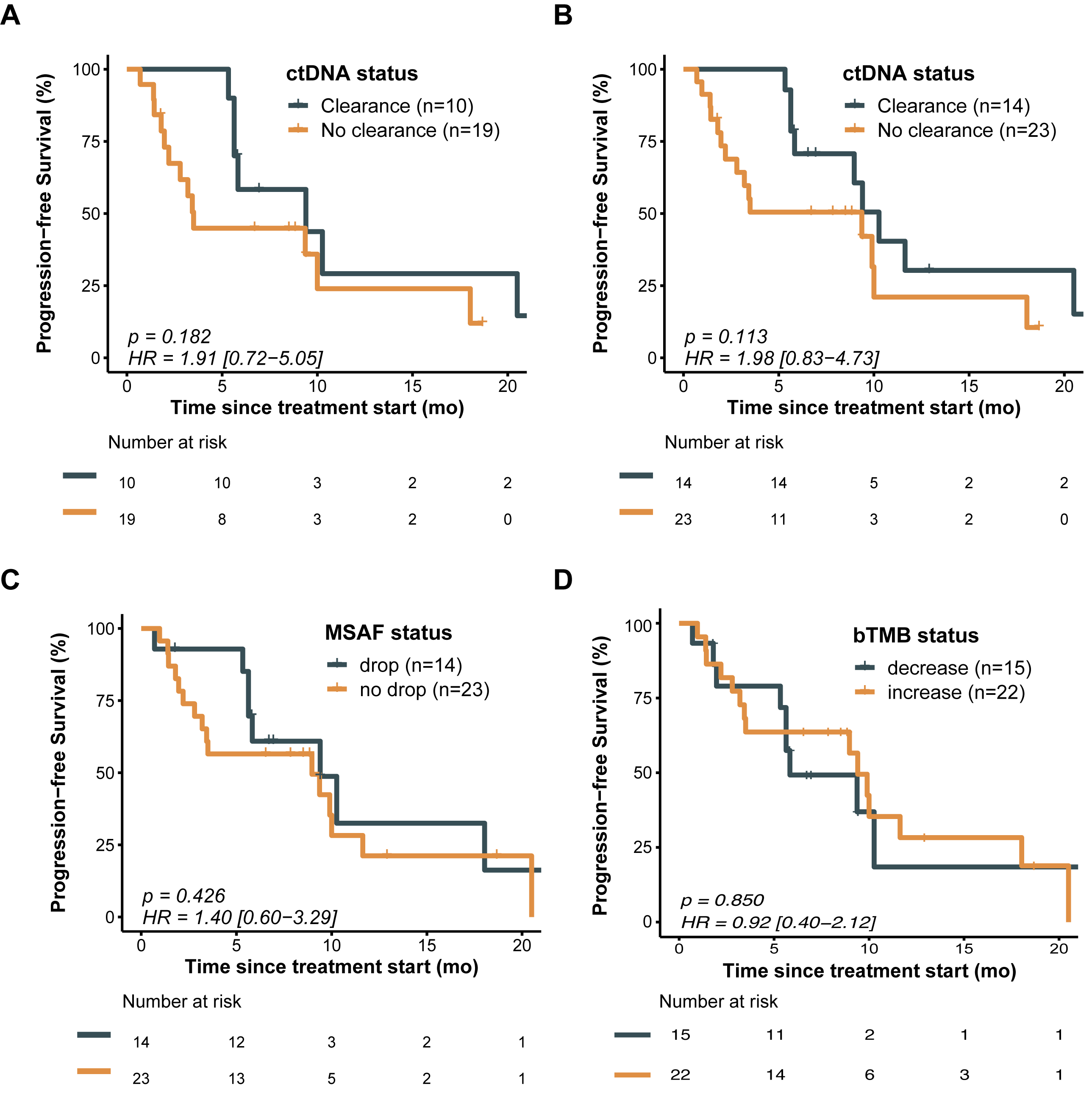


Fig. S4


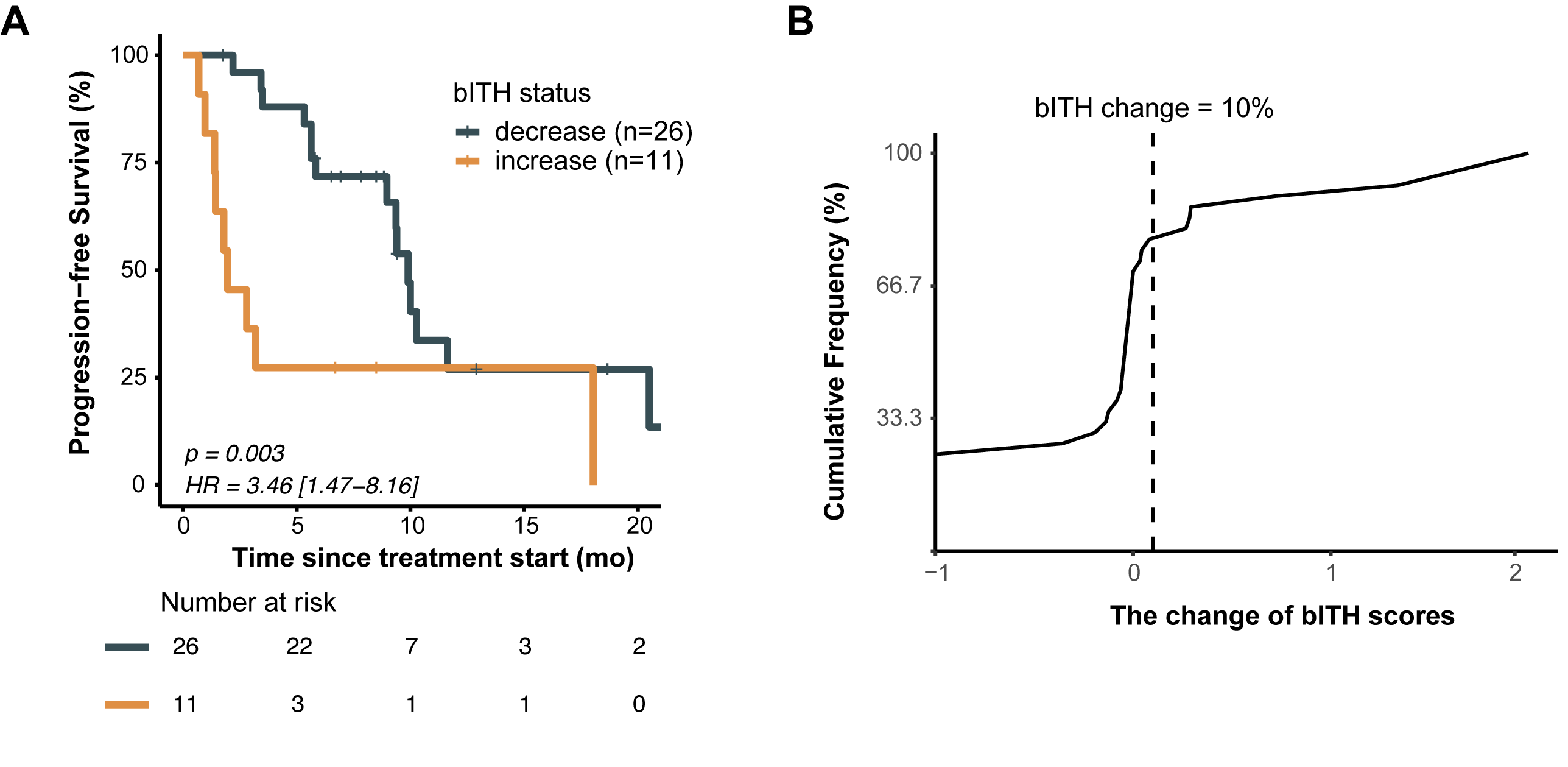


Fig. S5


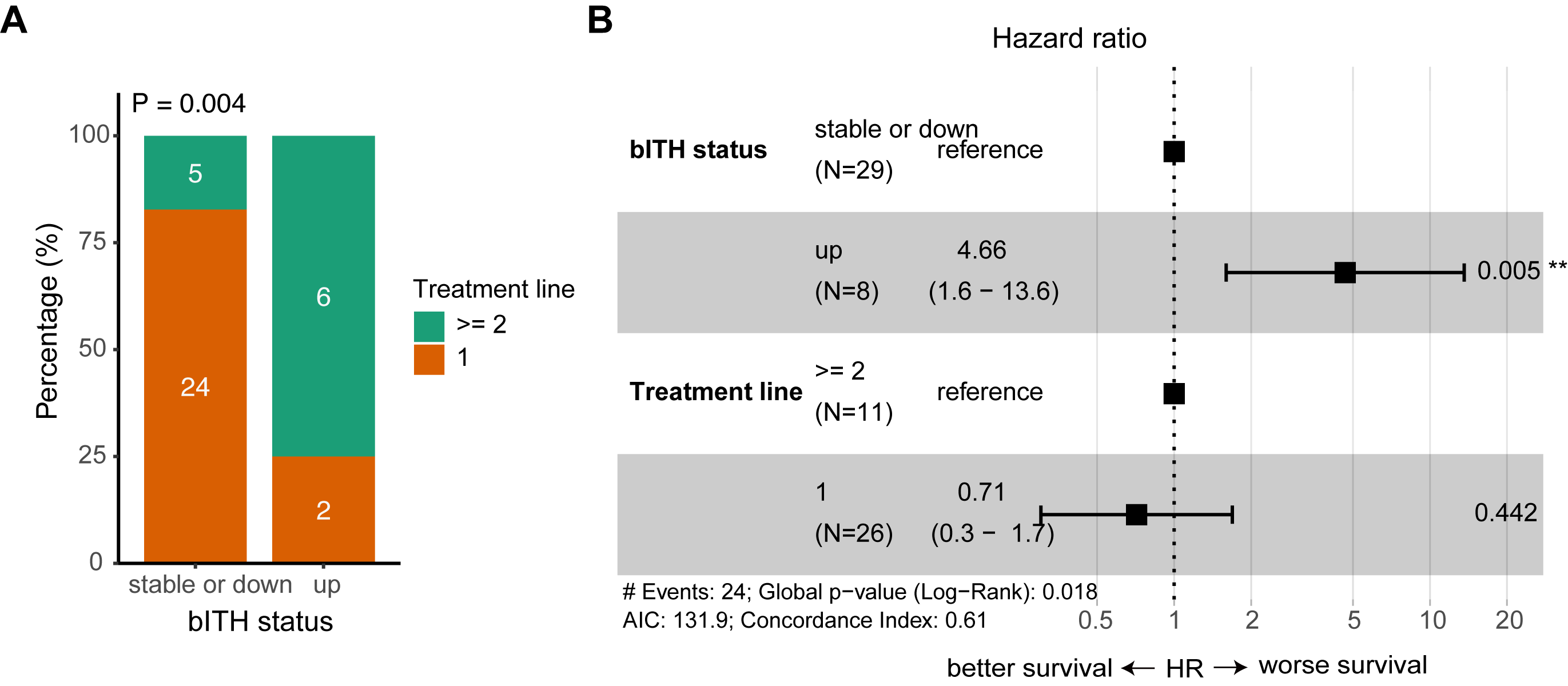

Supplement: Supplementary file 2 — Additional file 2: Fig. S1. Clinical outcomes of ICIs plus chemotherapy in the enrolled cohort. Fig. S2. The association of KEAP1 mutation at baseline with clinical outcomes of ICIs plus chemotherapy. Fig. S3. Kaplan-Meier curve for progression-free survival (PFS) according to ctDNA clearance status, MSAF change status, and bTMB change status. Fig. S4. The cutoff selection of bITH change. Fig. S5. The association of bITH change with progression-free survival of ICIs plus chemotherapy after adjusting to the treatment line of chemoimmunotherapy. [file 12916_2022_2444_MOESM2_ESM.docx]
